# Upcycling Milk Industry Byproducts into *Tenebrio molitor* Larvae: Investigation on Fat, Protein, and Sugar Composition

**DOI:** 10.3390/foods13213450

**Published:** 2024-10-29

**Authors:** Annalaura Brai, Cassia Neri, Franca Tarchi, Federica Poggialini, Chiara Vagaggini, Riccardo Frosinini, Sauro Simoni, Valeria Francardi, Elena Dreassi

**Affiliations:** 1 Department of Biotechnology, Chemistry and Pharmacy, University of Siena, via A. Moro, 53100 Siena, Italy; cassia.neri@student.unisi.it (C.N.); elena.dreassi@unisi.it (E.D.); 2 Research Centre for Plant Protection and Certification (CREA-DC), via di Lanciola 12/A, 50125 Firenze, Italy; franca.tarchi@crea.gov.it (F.T.); federic.poggialini@unisi.it (F.P.); chiara.vagaggini@student.unisi.it (C.V.); riccardo.frosinini@crea.gov (R.F.); sauro.simoni@crea.gov.it (S.S.); valeria.francardi@crea.gov.it (V.F.)

**Keywords:** edible insects, *Tenebrio molitor*, waste reduction, whey permeate, mozzarella whey, circular economy, proteins

## Abstract

Edible insects represent a growing sector of the food industry and have a low carbon footprint. Noteworthy, insects can upcycle different leftovers and byproducts into high-quality nutrients. Herein, the larvae of the edible insect *Tenebrio molitor* (TML) were fed using local milk industry byproducts. Mozzarella whey and whey permeate obtained in cheese production were used to formulate three alternative diets. Both byproducts are rich in sugars, in particular the disaccharide lactose and the monosaccharides glucose and galactose. Two of the three diets did not interfere with biometric data and vitality, while the use of whey permeate alone significantly reduced development. At the end of the trial, the proximate composition of TML was strongly affected, with an increased protein content of up to +7% and a favorable fat composition. The analysis of secondary metabolites revealed the accumulation of different compounds, in particular monounsaturated fatty acids (MUFAs), amino acids, and the disaccharide trehalose, essential for the correct larval development and pupation. In conclusion, the present study demonstrates that milk industry byproducts can be upcycled as feed for TML, maintaining an optimal nutrient composition and favorably increasing the protein content.

## 1. Introduction

As reported by the FAO, the global population is growing, increasing the demand for edible proteins. The market for alternative proteins is expected to grow at a compound annual growth rate (CAGR) of 13.0% from 2023 to 2030, reaching USD 40.7 billion globally [1]. Rapid urbanization, rising consumer aspirations, rising venture capital investments in the alternative protein sector, and the environmentally sustainable production and consumption of alternative proteins are the main factors driving this market’s growth [2]. The market’s expansion is, however, limited by the greater price of alternative proteins in comparison to traditional proteins and customers’ strong inclination toward animal-based products [3,4]. Because they can be produced at competitive prices, plant proteins have the potential to displace animal proteins in terms of market share [5]. Food consumption and diet supplements influence the demand for dairy and other animal proteins. Plant proteins are becoming more common in food products as the number of vegan and vegetarian consumers rises [5]. Furthermore, many natural products are produced using plant proteins. Overall, the market for protein and the demand for substitute protein ingredients are being driven by the expanding food sector because of rising consumer awareness and population [2].

In addition, addressing food security and protecting land and oceans are worldwide challenges brought by population expansion, climate change, and dietary changes [6]. As a result, there is growing interest in biodiverse and sustainable food systems. In this context, edible insects are a valuable and sustainable source of proteins. In an effort to identify alternatives to costly, overused, and environmentally damaging conventional protein sources, researchers have spent the last 20 years exploring the possibilities of edible insects as novel ingredients in high-value products. Because of their high protein level (average of 40% and up to 70% on a dry weight basis), mineral and vitamin contents, and favorable polyunsaturated-to-saturated fatty acid ratios, insects have consequently attracted increased attention in the food sector recently [7,8,9,10]. Differently from plants, insects contain all the essential amino acids and have higher digestibility [11]. According to Rumpold and Schluter (2013), edible insect proteins often satisfy the WHO’s necessary amino acid content standards [12]. In addition, the digestibility of insect proteins ranges from 76 to 38%, which is higher than that of plant-based proteins like peanuts and lentils (52%) and is only slightly lower than that of animal-based proteins like egg white and beef [7]. According to the EFSA, *Tenebrio molitor* larvae (TML) are the first edible insects to be deemed safe in this regard [13]. TML are abundant in polyunsaturated fatty acids (PUFAs) and peptides, having antibacterial and antihypertensive qualities, as seen from a nutraceutical perspective [14,15,16,17]. TML possess different bioactive peptides, endowed with antibacterial [18], anticancer [19], and antiadipogenic [20] activities. Many peptides generated in vivo after gastrointestinal digestion have reduced the blood pressure in hypertensive rats. Their capability of inhibiting angiotensin-converting enzyme (ACE), makes TML a functional food useful for the prevention of cardiovascular diseases [14]. ACE-inhibitory activity has been already observed for other foods, in particular bovine whey and muscle proteins of meat, fish, and invertebrates [21,22]. Several edible plants have mild ACE-inhibitory activity, in particular buckwheat, broccoli, potato, and spinach [23]. In contrast, seaweeds have potent ACE-inhibitory activity, but due to the maximum daily intake recommended by the EFSA, the consumption of seaweeds alone can be inadequate [24,25]. TML have an extraordinary capability to metabolize and accumulate active components from the rearing substrate; indeed, different agrifood byproducts were used to increase antioxidant activity and ameliorate the nutrient profile [26,27,28,29,30,31]. Moreover, because of their adaptability to various substrates, their growth can be accelerated by upcycling byproducts [32]. Agricultural leftovers such as fallen leaves or byproducts like tomato peels and seeds increased antioxidant compounds in TML [29]. Potato peel, corn cob, and rice bran combined with essential oil post-distillation residues boosted the mean weight and antioxidant activities of TML [33]. Dried potatoes, either by themselves or mixed with egg whites can speed up development, probably due to higher protein content [34]. Analogously, the high protein content of distillery byproducts increased the mean weight of TML [32]. In contrast, the essential oils of oregano, thyme, garlic, and caraway, and many secondary plant metabolites, especially terpenes, terpenoids, and certain aldehydes, were shown to be poisonous or repellent for TML [35,36,37].

Milk byproducts derived from cheese production have been used for centuries for the preparation of bakery products and animal feeds, due to their valuable nutrient properties, in particular, a high protein content [38]. Among milk proteins, whey proteins have also been of interest in the pharmaceutical and food industry. Milk proteins are recovered from milk by ultrafiltration, creating a byproduct known as milk permeate, rich in lactose, minerals, traces of proteins and fat, and vitamins, in particular vitamins of group B [39]. In the dairy industry, permeates are produced as leftovers from the processing of products like protein concentrated from whey or milk. According to the literature, permeates are composed of 2–7% protein, 76–86% lactose, 0–1% fat, and 8–11% ash [40]. Processing conditions vary significantly across suppliers leading to different varieties of permeate (acid, sweet, and milk).

Permeate was once thought to be waste or used to make animal feed, but with the recent shift in the economy toward a “zero waste” economy, these streams are now being studied for their potential use as raw materials or ingredients for products with increased added value. To valorize local byproducts and upcycle them into a valuable resource, we decided to study the possible use of mozzarella whey (MW) and whey permeate (MP) as TML feed.

## 2. Results and Discussion

### 2.1. Analysis of Byproducts

As reported in Table 1, both byproducts are mainly composed of carbohydrates, while differing in the content of proteins and fats. The data are in accordance with previously published data [39,41,42]. WP accounts for 0.98% of fats and 2.51% of proteins, as already reported [39,41]. The main components are carbohydrates, accounting for 85% of DW. The content of carbs in MW is lower than in WP, accounting for 65% of dry weight, in perfect agreement with Gernigon et al. [42]. Notably, fat accounts for 8% of weight, which is eight times higher than in WP.

The ^1^HNMR analysis of MW and WP revealed a different composition. MW was mainly composed of lactose and galactose, accounting for about 72% and 9% of the total components identified, followed by organic acids (10.73%) and amino acids (3.88%). Among the organic acids, the most abundant is lactate, followed by residual SCFAs such as propionate and butyrate. WP has a different composition of nutrients and secondary metabolites. Even in this case, lactose is the most abundant compound, followed by organic acids. Amino acids were not detected, due to their lower concentration in the whole extract. The sugar content in NMR analysis is higher with respect to previously published data and nutrient analysis [42,43] due to the filtration process, which excludes molecules with molecular weight higher than 3000 Da, in particular whey proteins. Galactose has been identified only in MW. The production of mozzarella cheese involves the use of thermophilic starters, such as Lactobacillus bulgaricus and Streptococcus thermophilus, which do not ferment galactose and hence keep it unaltered in the whey [42]. MW has a lower concentration of lactose with respect to WP, as a result of having the largest amounts of lactate and galactose, which are byproducts of lactose’s bioconversion [42].

### 2.2. Analysis of Survival Rate and Body Weight

Dairy byproducts have been used as feed supplements for different animals. In this regard, milk whey has been historically administered as a supplement for pigs, cows, and cattle [44]. Nowadays, whey permeate is still upcycled into animal feeds. Beom Jang et al. reported that the addition of whey permeate can also have beneficial effects, associated with the stimulation of the intestinal immune response and enterocyte proliferation [3]. Accordingly, nursery pigs grew most effectively with the addition of 13.6% whey permeate, increasing from 7 to 11 kg BW.

**Figure 1:**
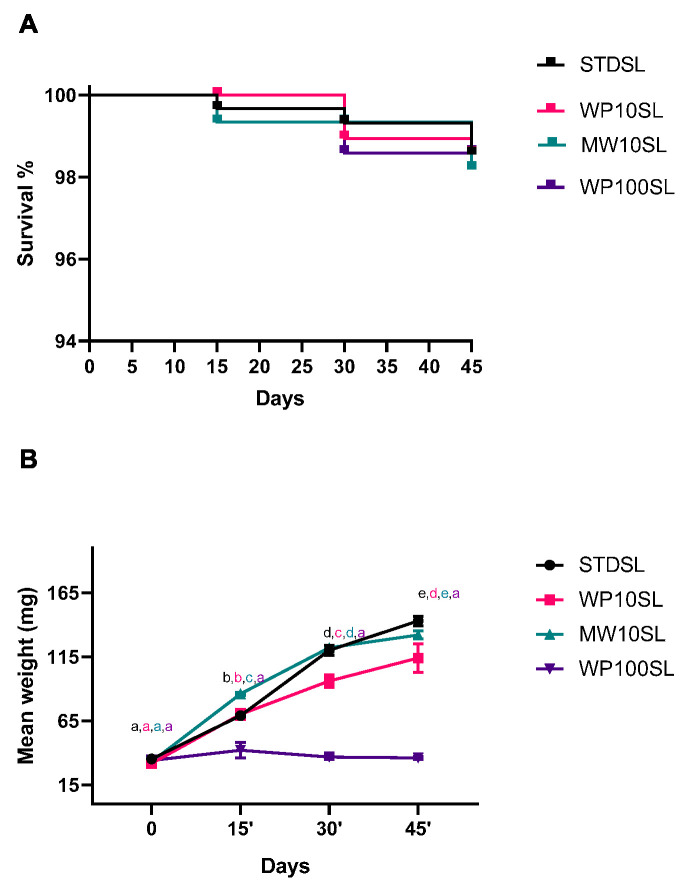
(**A**) Kaplan–Meier curve of *Tenebrio molitor* larvae fed on a standard diet or standard diet supplemented with dairy farm byproducts. Standard diet larvae (STDSL) were used as control. Whey permeate-supplemented larvae (10% *w*/*w*) (WP10SL); mozzarella whey-supplemented larvae (10% *w*/*w*) (MW10SL); whey permeate-supplemented larvae (100% *w*/*w*) (WP100SL). Curve comparison was analyzed by Mantel–Cox test (Χ^2^ = 0.1773, df = 3, *p* = 0.9134). (**B**) Mean body weight over time. Statistical analysis was performed with two-way ANOVA with different diets and time of rearing (15, 30, and 45 days) as fixed factors and Tukey’s post-hoc test. ^a–e^ Different superscript letters indicate a significant difference among the means; *p* < 0.05 by post-hoc Tukey’s test.

Mortality was analyzed during the experiment, and as shown in Figure 1A, no statistically significant differences (Mantel–Cox test, Χ^2^ = 0.1773, df = 3, *p* = 0.9134) were found between the control group, fed with a cereal based standard diet (STDSL), and groups supplemented with whey permeate or mozzarella whey at 10% w/w (WP10SL or MWSL), or entirely with whey permeate (WP100SL). This result was not surprising, as whey permeate and whey have been used as feed supplements for insects. In this regard, cheese whey has been used to feed black soldier fly larvae (BSFL); the replacement of 70% of the standard diet with cheese whey did not affect vitality [45]. In another study, Caltzontzin-Rabell et al. increased cheese whey up to 75%, without observing a mortality increase in BSFL even after storage [46]. The absence of increased mortality has already been reported for different agricultural substrates like fallen leaves, hemp, spent grains, and distillery byproducts [27,29,31,32,47]. Although a toxic impact was previously noted in insects treated with orange essential oil, no toxicity was seen in TML-fed orange albedo [26]. The removal of flavedo, which includes the volatile aromatic compounds, is likely what led to the high survival rates in this instance as well [48]. Processed wastes are typically well tolerated, even at high percentages. In fact, at concentrations ranging from 0 to 50%, carob pods and hemp waste did not affect survival rates [27,33].

On the contrary, different diets determined significant differences in the mean weight of the experimental groups over time [45,46]. The mean weight was analyzed with two-way ANOVA by considering time and feed as fixed factors. The effect of feeds was statistically significant (df = 3, F = 490.1, *p* < 0.0001), as well as the effect of time (df = 3, F = 743.6, *p* < 0.0001). As reported in Figure 1B, the mean weight increased over time in all the experimental groups, except for WP100SL, which probably due to the absence of adequate nutrient apport did not grow during the entire trial. On the contrary, replacing 10% of the standard diet with MW or WP maintains a mean weight comparable to or slightly below the control.

### 2.3. Analysis of Proximate Composition

The larvae’s bromatological analysis revealed significant changes in nutrient composition among the four groups (Table 2). The dry matter content was higher in the WP groups, while remaining comparable to the control in the MW10SL group. The proteins increased in all the experimental groups, reaching +7% in MW10SL and +3 and +2 in WP10SL and WP100SL groups. The crude protein content found in the control group is comparable to the data previously published using a conversion factor of 4.76 [49,50,51] The classical conversion factor of 6.25 overestimates protein, due to the chitin content, and data are higher than those obtained in the present study as well as in other studies already reported [49]. Byproducts can increase the protein content; interestingly, *Moringa oleifera* leaf supplement increased crude proteins by +9% [52]. The addition of chestnut shells increased crude proteins by +7.44% [50]. According to the higher quantity of crude proteins with respect to WP, the protein content was maximal in the WPSL group. A similar trend has been reported for *Moringa oleifera*-enriched diets [52]. Fat represents the most abundant nutrient in the control group. Only minimal differences were observed in the total fat content after 45 days of rearing. The total fat content decreased in a statistically relevant way in the MW10SL group, with a value reduced of 4% with respect to control. The carbohydrates increased with respect to control, in particular in the groups fed with whey permeate, with values up to +6%. The increased content of carbs is related to the high content of sugars in byproducts, in particular lactose and galactose. Considering other meats, TML have a protein content comparable to or higher than in lamb and pig, but lower than in beef and chicken [53]. In contrast, fat is higher than in beef and chicken, but lower than in lamb and pork [53]. Interestingly, the quality of fat is higher, accounting for more than 74% of UFAs. Considering the energy requirements, the FAO and WHO recommend a daily energy intake ranging from 2550 to 4000 kcal for a 75 kg man, based on the basal metabolic rate [54]. Athletes require high energy intake and a different intake level of nutrients. The recommended carbohydrate intake should be increased up to 12 g/kg of body weight, while proteins can reach up to 2.2 g/kg of body weight. Fat represents an important source of energy; in this case, the recommended value comprises between 20 and 35% of total fat intake [54,55,56]. Considering those values, TML can satisfy both normal and athletes’ requirements in terms of fat and proteins, while carbs are below the recommended range as usually observed for meats.

### 2.4. Analysis of Fat

**Figure 2:**
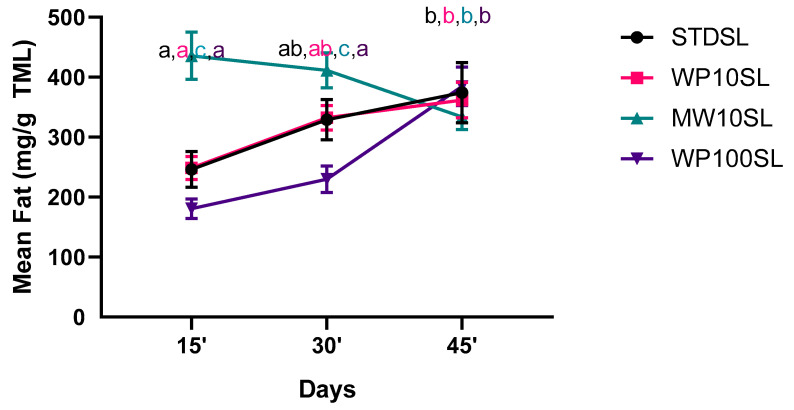
Fat (mg of fat/g of TML) over time. Results represent the mean ± SD of three experiments. Standard diet-supplemented larvae (STDSL) were used as control. Whey permeate-supplemented larvae (10% *w*/*w*) (WP10SL); mozzarella whey-supplemented larvae (MW10SL); whey permeate-supplemented larvae (100% *w*/*w*) (WP100SL). (Statistical analysis was performed with two-way ANOVA with different diets and time of rearing (15, 30, and 45 days) as fixed factors and Tukey’s post-hoc test.) ^a–c^ Different superscript letters indicate a significant difference among the means; *p* < 0.05 by post-hoc Tukey’s test.

The mean fat was determined over the experiment and analyzed with two-way ANOVA by considering time and diets as fixed factors. (Figure 2) The effect of diets was statistically significant (df = 2, F = 24.59, *p* < 0.0001), as well as the effect of time (df = 3, F = 28.16, *p* < 0.0001). The administration of the supplemented diets significantly modified the mean fat. After receiving an MW supplement rich in fat, the MWSL group immediately increased their fat composition, as we can observe after 15 days of treatment. The fat decreased over time, reaching values statistically comparable to the control after 45 days. Interestingly, after 15 days of treatment, when the fat reached the maximum value, MW10SL had a mean weight higher than 25% with respect to control, suggesting that a diet rich in fat can speed up the growth of TML during the first phases of development. In WP10SL, the fat remained comparable with STDSL, increasing over time. The WP100SL group had a similar trend, but the fat significantly increased at the end of the experiment.

Fat composition was thoroughly analyzed with both ^1^NMR and GC-FID. Despite being necessary for human nutrition, fat should only be ingested responsibly, because eating low-quality fat has been related to an increased risk of cardiovascular death [57,58,59]. As determined in NMR analysis, the main components of the lipidic extracts were triglycerides, phosphatidylcholine esters, and cholesterol. Interestingly, cholesterol content decreased in all the experimental groups with respect to control (30%, 20%, and 11% in WP10SL, WP100SL, and MW10SL, respectively). Diets had a strong impact on the quality of fatty acids. Even if the supplements have a low quality of fats (Table S1, Supporting Information), mainly composed of saturated fatty acids, TML maintained their typical fatty acid composition, rich in MUFAs and PUFAs. This is not surprising because TML synthesized de novo different fatty acids, in particular oleic, linoleic, and linolenic acids [60,61,62]. As reported in Table 3, oleic acid is the most abundant FA, varying between 38 and 42% of the whole FA content. Noteworthy, consuming oleic acid in food has been demonstrated to lower blood pressure and the risk of developing cardiovascular disease [63,64], suggesting that the consumption of TML may be beneficial. Palmitic and stearic acids were the most abundant SFA, while oleic and linoleic were the most abundant MUFA and PUFA, in agreement with previous studies [29,47,65,66]. Palmitic acid varied, on average, between 14 and 20%, while linoleic acid varied between 19 and 22%. Notably, the quantity of UFA statistically increased in MW10SL and WP100SL, while it slightly decreased in WP10SL.

SFA may be related to increased risk of cardiovascular disease. C14:0 and C16:0 are among the most atherogenic SFAs, while C18:0 is considered to be thrombogenic but neutral in terms of atherogenicity. In order to evaluate the potential risks, the nutritional indexes associated with increased cardiovascular disease risks were calculated. The atherogenicity index (AI) proposed by Southgate [67] represents the ratio between the total amount of SFAs and the total amount of unsaturated fatty acids (UFAs). The calculated values were comparable to the control group and resulted in being between 0.47 and 0.54. The values are similar to those reported for brown and green seaweeds [68,69], crops, and chicken [70], while fast foods like beef burgers and Margherita pizza have AIs of 0.99 and 1.99, respectively. Regarding the thrombogenicity index, which represents the probability of forming clots in the arteries, it is calculated as the ratio between the saturated thrombogenic fatty acids and the unsaturated anti-thrombogenic fatty acids. We calculated values of about 0.6 in MW10SL and WP100SL, slightly below the control group, while the IT increased in the WP10SL group, with a value of 0.8. Even in this case, the value is comparable to the range determined for brown seaweeds [68] and chicken [71] while lower than meat like lamb and rabbit (1.2 and 1.1, respectively), or processed foods like Margherita pizza (2.42), beef burger (1.40), and Bologna sausages (1.55) [70,72]. Although no organization has yet to establish the suggested values for IA and IT, consuming food with a lower IA and IT has superior nutritional quality and may reduce the risk of coronary heart disease [64]. The health-promoting index (HPI) assesses the nutritional value of dietary fat, and it is mainly used for dairy products such as milk and cheese, with values comprised between 0.3 and 0.7 [73]. The calculated values are significantly higher, with ranges from 1.9 to 2.1. The favorable fatty acid composition coupled with decreased cholesterol concentration makes the fat quality particularly promising and ameliorates the quality concerning the control group.

### 2.5. Analysis of Polar Secondary Metabolites

To determine the modification of the hydrophilic secondary metabolites induced by diets, TML were subjected to methanol–water extraction and analyzed by NMR. The superimposition of the spectra highlighted the main differences in the aminoacidic concentration and sugar composition.

**Figure 3:**
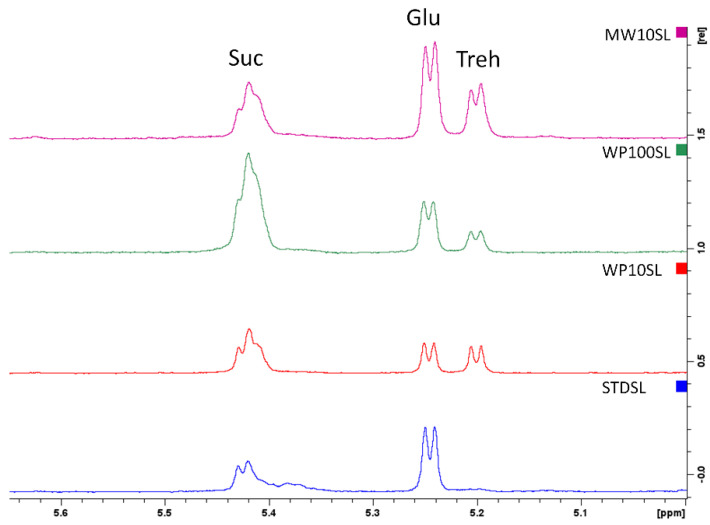
Superimposition of the ^1^HNMR spectra of the aqueous extracts of *Tenebrio molitor* larvae after 45 days of treatment. Standard diet-supplemented larvae (STDSL, blue) were used as control. Whey permeate-supplemented larvae (10% *w*/*w*) (WP10SL, red); mozzarella whey-supplemented larvae (MW10SL, violet); whey permeate-supplemented larvae (100% *w*/*w*) (WP100SL, green). Suc (sucrose), Glu (glucose), Treh (Trehalose).

Firstly, the sugar composition was investigated. As reported in the Figure 3, the control group fed with the standard diet had a high concentration of glucose and sucrose, while the signal of trehalose, already reported as the main disaccharide for the larvae fed with wheat bran, was absent [74]. Trehalose is synthesized from glycogen and plays a crucial role in the growth and development of insects. Increased concentration of trehalose is necessary for the chitin synthesis process’s requirement for energy and glucose [75]. In the WP10SL and WP100SL groups fed with whey permeate, the signal of trehalose is visible at 5.22 ppm, and the concentration of sucrose increased in the group that received the highest concentration of WP (+36.56%), in accordance with spectrophotometric data reported in the Table 2. In MWSL extract, the trehalose signal is visible, and the concentration of glucose and sucrose increased by +5.39 and 29.66%, respectively. Even if present as the major component of the supplements, lactose was not found in TML extracts.

Amino acids and organic acids were quantified in the whole extracts, and the results are shown in Table S2. Even if, due to the absence of gastrointestinal and acidic hydrolysis, the amino acids did not reflect the real bioavailable aminoacidic portion, we found that the MW10SL group has the higher quantity of amino acids, reflecting the higher protein content found. The most abundant amino acids were proline, glutamate, tyrosine, and arginine, according to data already reported [50,74]. Among the essential amino acids, methionine and histidine were not quantified due to an S/N ratio < 10.

## 3. Materials and Methods

### 3.1. Materials

Materials were purchased from Sigma-Aldrich S.r.l. (Milan, Italy) and used without further purification.

### 3.2. Design of the Experiment

The experimental protocols were performed according to the guidelines of the European Directive (2010/63/EU) on the protection of animals used for scientific purposes. Larvae used for the experiments were reared at the Research Centre for Plant Protection and Certification (CREA-DC Florence, Italy) on a standard diet previously published and used as a control [76]. STD was composed of brewer’s yeast (0.5%), wheat flour (49.75%), and oats (49.75%). Mozzarella whey was purchased at a local market. Vaccine whey permeate was kindly provided by the “Mukki” farm (Firenze, Italy). The water was removed using a rotary evaporator. Moisture content was finally reduced to 10% w/w by drying wastes under vacuum at 20 °C for 48 h. Dried wastes were finely pulverized using a Bosch food processor, TSM6A013B (Bosh, Gerlingen-Schillerhoehe Germany), and sieved through a 500 µm Endecotts test sieve (Endecotts Ltd., London, UK); 10% w/w of the standard diet described above was replaced with mozzarella whey or whey permeate, or completely replaced with whey permeate obtaining the corresponding supplemented feeds.

**Figure 4:**
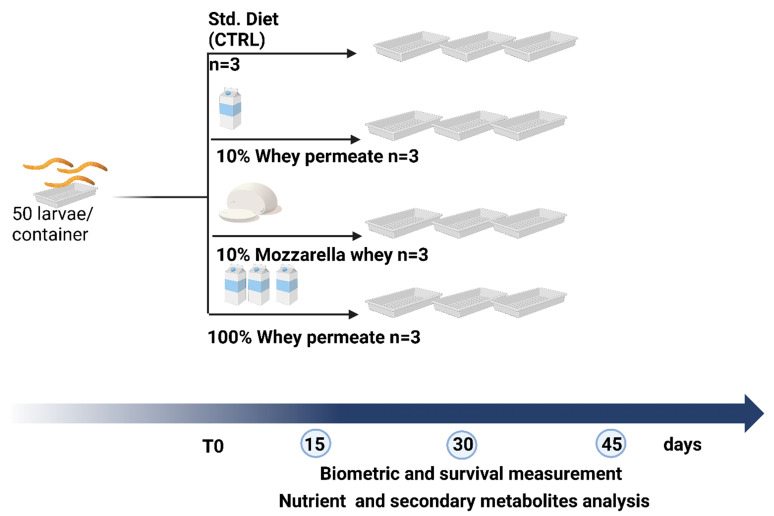
Experimental design.

Thirty-day-old larvae with lengths comprising between 15 and 20 mm were used for the experiment. Larvae were allocated into plastic containers (50 TML per container) and randomly divided into four experimental groups that received the cereal-based Std. Diet (STDSL) [76] or the alternative diets (WP10SL, WP100SL, or MW10SL) (Figure 4). Each experiment was performed in triplicate. Larvae were maintained in semi-dark conditions in a climate room at 27 ± 1 °C and RH (relative humidity) 40–50% [77]. At 15, 30, and 45 days, larvae were counted, weighed, freeze-dried using an Edwards MOD freeze-dryer (Edward and Co., Ltd., London, UK), and kept at −20 °C until they were used for analysis.

### 3.3. Extraction of Polar Metabolites

TML were extracted using a previously published protocol [65]. In detail, 1 g of dried insects were extracted by adding 10 mL of a MeOH/H_2_O mixture and homogenizing with an IKA Labortechnik T25 basic (IKA Werke GmbH & Co., Staufen, Germany). After centrifugation for 5 min supernatant and pellet were separated and the solvent was removed. The procedure was repeated two times, and the solvent was removed under N_2_ flow. The samples were frozen at −20 °C before analysis.

### 3.4. Total Lipid Content Determination

Lipophilic extracts were prepared using the Folch method [78]. Each sample (1 g) was exactly weighed and placed in a glass centrifuge tube with 10 mL of CHCl_3_/CH_3_OH mixture (2:1 *v*/*v*) and homogenized for 5 min using an IKA Labortechnik T25 basic (IKA WERKE GmbH & Co., Staufen, Germany).

The mixture was centrifuged at 6000× *g* for 5 min. The CHCl_3_ layer was separated, and the extraction repeated. The organic layer was filtered and washed with 10 mL of KCl 0.7% solution, washed at the interface twice with the “upper phase” (prepared from CHCl_3_/CH_3_OH/H_2_O (53:27:20 *v*/*v*) mixture), and dried under vacuum.

The extract was weighed to calculate fat percentage and then used for FA analysis. The same fat extraction procedure was adopted for byproducts starting from 1 g of material.

### 3.5. Analysis of Nutrients

The oven-drying method was adopted to determine the moisture. In detail, 1 g of each sample was weighed and dried over 14–16 h at 105 °C. The moisture was determined by subtracting the initial weight from the final weight.

The nitrogen content was determined using the Kjeldahl method starting from 1.0 g of sample. The crude protein content was calculated using a conversion factor of 6.25 for rearing substrates and 4.76 for TML [49].

Energy values were calculated using the following conversion factors: 3.87 kcal/g for carbohydrates, 9.02 kcal/g for fats, and 4.27 kcal/g for proteins.

### 3.6. NMR Analysis

Dried lipophilic extracts (20 mg) were solubilized in 0.7 mL of CDCl_3_, containing 0.03% (*v*/*v*) tetramethylsilane (Sigma Aldrich, Darmstadt, Germany). Polar extracts were solubilized in 1.0 mL of 400 mM phosphate buffer/D_2_O (pH 7) containing 3-(trimethylsilyl)propionic acid sodium salt (TSP) 1 mM as a standard. The samples were filtered prior analysis. Mono-dimensional ^1^HNMR spectra were recorded at T = 298 K using a Bruker Advance DPX 400 MHz spectrometer (Bruker Biospin, Billerica, MA, Germany). ^1^HNMR of lipophilic extracts were acquired with a single pulse experiment, with a 90° excitation pulse, 4 dummy scans, 3 s relaxation delay, and 16 scans. The spectra of the polar extracts were analyzed using the Bruker zgpr sequence. ^1^HNMR spectra were assigned by comparison with the published data [50,74] and Chenomx NMR suite v 11 (Chenomx Inc., Edmonton, AB, Canada) and quantified using the same software.

### 3.7. Fatty Acid Determination

The fatty acid methyl esters (FAMEs) were obtained by reaction with 14% BF_3_ methanolic solution. The lipophilic extract (5 mg) was reacted with 1 mL of BF_3_ methanolic solution at 90 °C for 1 h and then, after cooling, the FAMEs were extracted with 1 mL of n-hexane and used for GC analysis.

The GC-FID analyses were performed with a GC-FID (Perkin Elmer Clarus 500 GC) as previously reported [79]. Temperatures of injector and detector were 230 and 280 °C, respectively. 1 mL/min of helium was employed as a carrier; 1 µL aliquots were injected and a split ratio of 1:10 was used. An SPTM-2380 fused silica capillary column (60 m, 0.25 mm I.D., 0.2 µm film thickness) provided by Supelco (Bellefonte, PA, USA) was used. Oven temperature was programmed from 100 to 240 °C at 10 °C/min, then to 260 at 5 °C/min, and finally maintained at 260 °C for 10 min. The injector temperature was set at 230 °C.

Data acquisition was carried out using Perkin Elmer TotalChrom Navigator software v 6.3.1. The identification of various FAs was carried out by comparison with retention times of the available standards, and on the basis of elution provided in the literature [47].

### 3.8. Lipid Quality Indices Determination

Indices of thrombogenicity (IT) and atherogenicity (AI) were calculated based on FA composition as previously described [67] using the following formulas:

$$\mathrm{IT}=\frac{\left(C14:\;0\right)+\left(C16:\;0\right)+\left(C18:\;0\right)}{\left(0.5\times \mathrm{MUFA}\right)+\left(0.5\times \mathrm{Cn}6\right)+\left(3\times \mathrm{Cn}3\right)+\left(n3\times \mathrm{Cn}6-1\right)}$$

$$\mathrm{AI}=\frac{\left(C12:\;0\right)+\left(4\times C14:\;0\right)+\left(C16:0\right)}{\mathrm{PUFA}\;\left(\mathrm{Cn}6+\mathrm{Cn}3\right)+\mathrm{MUFA})}$$

Hypocholesterolemic/hypercholesterolemic (HH) ratio was calculated as reported by Silva et al. [80] using the following equation:

$$\mathrm{HH}=\frac{\mathrm{cis}-C18:1+\Sigma \mathrm{PUFA}}{C12:0+C14:0+C16:0}$$

The health-promoting index (HPI) was calculated as reported by Chen et al. [73] with the following formula:

$$\mathrm{HPI}=\frac{\Sigma \mathrm{PUFA}}{C12:0+\left(4\times C14:0\right)+C16:0}$$

### 3.9. Statistical Analysis

All determinations were run at least in triplicate and data were expressed as mean ± standard deviations (SD). Statistical analysis was performed with GraphPad Prism 8.2 (GraphPad Software, La Jolla, CA, USA). The data’s homoscedasticity and normality were confirmed with the Brown–Forsythe and Shapiro–Wilk tests.

The Kaplan–Meier graph was analyzed with the Mantel–Cox test, and data with *p* ≤ 0.05 were considered significant.

One-way analysis of variance (ANOVA) was performed to compare proximate composition, fatty acids, and polar metabolites between the groups. Significant differences between STD and supplemented diets are indicated with *** *p* ≤ 0.001, vs. STD (ANOVA), ** *p* ≤ 0.01 vs. STD, and * *p* ≤ 0.05 vs. STD. Two-way ANOVA with different diets and times of rearing (15, 30, and 45 days) as fixed factors was used to examine the mean increase in body weight and the fat quantity over time.

## 4. Conclusions

In conclusion, this work demonstrates that, if administered in limited quantities, milk industry byproducts can be favorably upcycled as TML feed. While the mean weight and survival rate remained unchanged, the nutrient composition significantly differed from the control, exhibiting an increased protein value. The secondary metabolites were analyzed, resulting in higher levels of MUFA, trehalose, and sucrose, but reduced cholesterol.

## Figures and Tables

**Table 1:** Analysis of nutrients in mozzarella whey (MW) and whey permeate (WP): proteins, carbohydrates (carbs), and fats ^#^.

Nutrients (% DW)	MW	WP
Fats	8.12 ± 1.22 ^b^	0.98 ± 0.12 ^a^
Proteins	10.87± 1.98 ^b^	2.51 ± 0.37 ^a^
Carbs	65.26 ± 2.25 ^a^	84.53 ± 1.25 ^b^

^#^ Results represent the mean ± SD of three experiments. Mozzarella whey (MW), whey permeate (WP). Values are expressed as g per 100 g of dry weight. ^a–b^ Different superscript letters indicate a significant difference among the means in each column; *p* < 0.05 by post-hoc Tukey’s test.

**Table 2:** Analysis of the proximate composition of *Tenebrio molitor* larvae reared on standard and supplemented feeds after 45 days of rearing ^#^.

	STDSL ± SD	WP10SL ± SD	MW10SL ± SD	WP100SL ± SD
Proteins (% DW)	35.97 ± 0.73 ^a^	39.17 ± 1.23 ^b^	43.08 ± 0.65 ^c^	38.11 ± 1.07 ^b^
Fat (% DW)	37.42 ± 1.40 ^b^	36.18 ± 1.10 ^b^	33.36 ± 1.91 ^a^	38.47 ± 2.18 ^b^
Carbs (% DW)	6.56 ± 0.55 ^a^	12.42 ± 0.69 ^c^	10.41± 0.83 ^b^	12.95± 0.98 ^c^
Energy (kcal/100 g)	516.83 ± 17.87	541.66 ± 15.91	525.14 ± 22.25	559.84 ± 26.09

^#^ Results represent the mean ± SD of three experiments. Standard diet-supplemented larvae (STDSL) were used as control. Whey permeate-supplemented larvae (10% *w*/*w*) (WP10SL); mozzarella whey-supplemented larvae (10% *w*/*w*) (MW10SL); whey permeate-supplemented larvae (100% *w*/*w*) (WP100SL). ^a–c^ Different superscript letters indicate a significant difference among the means in each column, *p* < 0.05 by post-hoc Tukey’s test.

**Table 3:** Analysis of FA composition and lipid quality indices of *Tenebrio molitor* larvae reared on standard and supplemented feeds after 45 days of rearing ^#^.

FA (mol %)								
Diets	STDSL	±SD	WP10SL	±SD	MW10SL	±SD	WP100SL	±SD
Capric acid 10:0	0.02	0.00	0.05	0.02	0.03	0.01	0.03	0.00
Lauric acid 12:0	0.75	0.01	0.97	0.01	0.49	0.08	0.57	0.00
Tridecylic acid 13:0	0.06	0.01	0.03	0.00	0.06	0.01	0.01	0.00
Myristic acid 14:0	5.47	0.47	4.04	0.33	5.58	0.05	5.21	0.04
14:1, 11	0.38	0.02	0.79	0.21	1.11	0.10	1.00	0.02
14:2n-3 acid	0.15	0.02	0.13	0.00	0.11	0.01	0.12	0.00
Myristoleic acid 14:1n-5	0.06	0.01	0.07	0.01	0.01	0.00	0.01	0.00
Pentadecylic acid 15:0	0.02	0.00	0.04	0.03	0.07	0.00	0.02	0.00
Palmitic acid 16:0	14.78	0.77 ^a^	20.36	0.07 ^b^	13.34	0.18 ^a^	14.58	0.43 ^a^
16:1n-5 acid	1.32	0.13	1.35	0.04	1.30	0.06	1.61	0.03
Palmitoleic acid16:1n-7	1.69	0.08	1.47	0.00	1.44	0.00	1.47	0.02
16:2n-4	0.08	0.01	0.05	0.00	0.07	0.00	0.03	0.00
Margaric acid 17:0	0.23	0.00	0.14	0.00	0.15	0.01	0.16	0.01
17:1 acid	0.26	0.02	0.39	0.00	0.12	0.01	0.29	0.01
Stearic acid 18:0	4.72	0.43	4.35	0.01	4.36	0.40	3.23	2.16
Oleic acid 18:1n-9	44.07	0.08 ^a^	42.01	0.06 ^a^	48.54	0.35 ^b^	47.00	1.06 ^b^
*α*-Linoleic acid 18:2n-6	21.94	2.01 ^b^	20.26	0.01 ^a^	18.91	0.58 ^a^	20.43	0.43 ^a^
Arachidic acid 20:0	0.15	0.01	0.23	0.01	0.17	0.01	0.24	0.00
*α*-Linolenic acid18:3n-3	0.23	0.01	0.14	0.01	0.15	0.01	0.16	0.05
Eicosenoic acid 20:1n-9	0.09	0.01	0.19	0.00	0.07	0.00	0.09	0.01
20:2n-6 acid	3.54	0.84	2.96	0.42	3.90	0.02	3.75	0.07
SFA	26.04	0.02 ^b^	30.12	0.21^c^	24.18	0.73 ^a^	23.92	1.69 ^a^
MUFA	47.88	0.34 ^b^	46.25	0.19 ^a^	52.59	0.19 ^c^	51.47	1.12 ^c^
PUFA	26.08	1.17 ^b^	23.63	0.40 ^a^	23.22	0.54 ^a^	24.62	0.56 ^a^
SFA/UFA	0.35	0.02 ^a^	0.43	0.00 ^b^	0.32	0.01 ^a^	0.31	0.03 ^a^
AI	0.51	0.04 ^ab^	0.54	0.02 ^b^	0.48	0.01 ^ab^	0.47	0.00 ^a^
TI	0.66	0.03 ^a^	0.80	0.01 ^b^	0.60	0.02 ^a^	0.59	0.06 ^a^
HPI	1.98	0.16 ^a^	1.87	0.07 ^a^	2.10	0.05 ^b^	2.11	0.01 ^b^

^#^ Results represent the mean ± SD of three experiments. Standard diet-supplemented larvae (STDSL) were used as control. Whey permeate-supplemented larvae (10% *w*/*w*) (WP10SL); mozzarella whey-supplemented larvae (10% *w*/*w*) (MW10SL); whey permeate-supplemented larvae (100% *w*/*w*) (WP100SL). Atherogenicity index (AI); thrombogenicity index (TI); health-promoting index (HPI). ^a–c^ Different superscript letters indicate a significant difference among the means in each column, *p* < 0.05 by post-hoc Tukey’s test.

## Data Availability

The original contributions presented in the study are included in the article/Supplementary Materials, further inquiries can be directed to the corresponding author.

## Supplementary Materials

The following supporting information can be downloaded at: https://www.mdpi.com/article/10.3390/foods13213450/s1, Table S1. Analysis of FA composition of mozzarella whey and milk permeate; Table S2. Analysis of selected polar secondary metabolites of *Tenebrio molitor* larvae reared on standard and supplemented feeds after 45 days of rearing.
