# Supplementary material for: Upcycling Milk Industry Byproducts into Tenebrio molitor Larvae: Investigation on Fat, Protein, and Sugar Composition
Source: Foods. 2024 Oct 29;13(21):3450. doi: 10.3390/foods13213450 (PMC11545053; doi:10.3390/foods13213450)
Supplement: Supplementary file 1 [file foods-13-03450-s001.zip › foods-3248921-supplementary.pdf]

Upcycling milk industry by-products into *Tenebrio molitor* mealworms: investigation on fat, protein and sugar composition

Annalaura Brai[1*], Cassia Neri[1], Franca Tarchi[2], Federica Poggialini[2], Chiara Vagaggini[2], Sauro Simoni[2], Valeria Francardi[2] and Elena Dreassi[1]

1 Department of Biotechnology, Chemistry and Pharmacy, University of Siena, via A. Moro - 53100 - Siena, Italy
2 Research Centre for Plant Protection and Certification (CREA-DC), via di Lanciola 12/A, 50125 Firenze, Italy

* **Correspondence:** Dr. Annalaura Brai, Department of Biotechnology, Chemistry and Pharmacy, University of Siena, via A. Moro - 53100 - Siena, Italy E-mail: annalaura.brai@unisi.it.

Keywords: edible insects; *Tenebrio molitor*; waste reduction; whey permeate; mozzarella whey; circular economy; proteins

Contents

Table S1. Analysis of FA composition of mozzarella whey and milk permeate....................................S3

Table S2. Analysis of selected polar secondary metabolites of *Tenebrio molitor* larvae reared on standard and supplemented feeds after 45 days of rearing S4

Table S1. Analysis of FA composition of mozzarella whey and milk permeate[#]

Fatty acid composition (%)				
Diets	**WP**	**±SD**	**MW**	**±SD**
Capric acid 10:0	1.48	0.16	1.68	0.66
Lauric acid 12:0	1.76	0.47[a]	4.24	0.53[b]
Tridecylic acid 13:0	0.00	0.00[a]	0.15	0.01[b]
Myristic acid 14:0	10.63	0.18[a]	18.10	0.31[b]
Myristoleic acid 14:1n-5	0.00	0.00	0.00	0.00
14:2n-3 acid	0.00	0.00[a]	0.38	0.02[b]
Pentadecylic acid 15:0	0.00	0.00[a]	0.72	0.00[b]
Palmitic acid 16:0	49.93	1.28	52.54	1.00
16:1n-5 acid	0.00	0.00[a]	0.61	0.06[b]
Palmitoleic acid16:1n-7	0.00	0.00[a]	0.73	0.02[b]
16:2n-4	0.00	0.00[a]	0.97	0.02[b]
Margaric acid 17:0	0.00	0.00[a]	0.10	0.03[b]
17:1 acid	0.00	0.00[a]	0.11	0.01[b]
Stearic acid 18:0	24.96	0.85[b]	16.95	0.31[a]
Oleic acid 18:1n-9	4.12	0.40[b]	0.45	0.10[a]
α-Linoleic acid 18:2n-6	0.60	0.06[b]	0.00	0.00[a]
Arachidic acid 20:0	0.00	0.00[a]	0.26	0.00[b]
α-Linolenic acid18:3n-3	0.00	0.00	0.00	0.00
Eicosenoic acid 20:1n-9	0.00	0.00	0.02	0.01
20:2n-6 acid	4.14	0.40[b]	0.14	0.04[a]

[#] Results represent the mean ± SD of three experiments. Whey permeate (WP), Mozzarella whey (MW). [abc] Different superscript letters indicate a significant difference among the means in each column, $p < 0.05$ by post hoc Tukey's test.

Table S2. Analysis of selected polar secondary metabolites of *Tenebrio molitor* larvae reared on standard and supplemented feeds after 45 days of rearing, data are reported in mg/g of DW [#]

	**STDSL**	**±SD**	**WP10SL**	**±SD**	**MW10SL**	**±SD**	**WP100SL**	**±SD**
Aminoacids								
Alanine	0.864	0.093	0.857	0.070	4.541	0.293	0.749	0.065
Arginine	2.051	0.198	2.497	0.140	3.889	0.314	1.957	0.091
Glutamate	4.090	0.317	5.383	0.247	8.299	0.398	4.026	0.322
Glutamine	1.595	0.095	1.708	0.041	2.137	0.227	1.853	0.197
Glycine	0.897	0.057	0.659	0.046	6.231	0.597	1.252	0.104
**Isoleucine**	1.278	0.126	1.646	0.131	1.957	0.175	2.029	0.196
**Leucine**	1.240	0.107	0.863	0.073	0.594	0.080	1.056	0.088
**Phenylalanine**	0.938	0.096	0.520	0.063	0.743	0.049	0.388	0.041
Proline	8.141	0.234	6.482	0.419	7.656	0.349	8.475	0.469
**Threonine**	0.420	0.051	0.268	0.015	0.462	0.057	0.448	0.034
Tyrosine	2.471	0.143	4.108	0.394	4.062	0.274	1.534	0.120
**Valine**	1.564	0.106	1.040	0.098	2.299	0.271	1.466	0.097
Organic acids								
Acetate	0.021	0.001	0.028	0.000	0.065	0.028	0.017	0.009
Formate	0.003	0.000	0.008	0.001	0.010	0.008	0.001	0.003
Lactate	0.016	0.000	0.021	0.001	0.019	0.018	0.003	0.016

[#] Results represent the mean ± SD of three experiments. Standard diet supplemented larvae (STDSL) were used as control, Whey permeate-supplemented larvae (10% w/w) (WP10SL), Mozzarella whey supplemented larvae (MW10SL), Whey permeate-supplemented larvae (100% w/w) (WP100SL). [abc] Different superscript letters indicate a significant difference among the means in each column, $p < 0.05$ by post hoc Tukey's test.
